# Sensor Histidine Kinase NarQ Activates via Helical Rotation, Diagonal Scissoring, and Eventually Piston-Like Shifts

**DOI:** 10.3390/ijms21093110

**Published:** 2020-04-28

**Authors:** Ivan Gushchin, Philipp Orekhov, Igor Melnikov, Vitaly Polovinkin, Anastasia Yuzhakova, Valentin Gordeliy

**Affiliations:** 1 Research Center for Molecular Mechanisms of Aging and Age-Related Diseases, Moscow Institute of Physics and Technology, 141700 Dolgoprudny, Russia; 2 Institute of Personalized Medicine, Sechenov University, 119146 Moscow, Russia; 3 Institute of Biological Information Processing (IBI-7: Structural Biochemistry), Forschungszentrum Jülich, 52428 Jülich, Germany; 4 European Synchrotron Radiation Facility, 38000 Grenoble, France; 5 Institut de Biologie Structurale J.-P. Ebel, Université Grenoble Alpes-CEA-CNRS, 38000 Grenoble, France; 6 JuStruct: Jülich Center for Structural Biology, Forschungszentrum Jülich, 52428 Jülich, Germany

**Keywords:** two-component systems, histidine kinase, receptor, transmembrane signaling, X-ray crystallography, molecular dynamics

## Abstract

Membrane-embedded sensor histidine kinases (HKs) and chemoreceptors are used ubiquitously by bacteria and archaea to percept the environment, and are often crucial for their survival and pathogenicity. The proteins can transmit the signal from the sensor domain to the catalytic kinase domain reliably over the span of several hundreds of angstroms, and regulate the activity of the cognate response regulator proteins, with which they form two-component signaling systems (TCSs). Several mechanisms of transmembrane signal transduction in TCS receptors have been proposed, dubbed (swinging) piston, helical rotation, and diagonal scissoring. Yet, despite decades of studies, there is no consensus on whether these mechanisms are common for all TCS receptors. Here, we extend our previous work on *Escherichia coli* nitrate/nitrite sensor kinase NarQ. We determined a crystallographic structure of the sensor-TM-HAMP fragment of the R50S mutant, which, unexpectedly, was found in a ligand-bound-like conformation, despite an inability to bind nitrate. Subsequently, we reanalyzed the structures of the ligand-free and ligand-bound NarQ and NarX sensor domains, and conducted extensive molecular dynamics simulations of ligand-free and ligand-bound wild type and mutated NarQ. Based on the data, we show that binding of nitrate to NarQ causes, first and foremost, helical rotation and diagonal scissoring of the α-helices at the core of the sensor domain. These conformational changes are accompanied by a subtle piston-like motion, which is amplified by a switch in the secondary structure of the linker between the sensor and TM domains. We conclude that helical rotation, diagonal scissoring, and piston are simply different degrees of freedom in coiled-coil proteins and are not mutually exclusive in NarQ, and likely in other nitrate sensors and TCS proteins as well.

## 1. Introduction

Two-component systems (TCSs) are a large family of signaling systems found mostly in bacteria and archaea that recognize a variety of environmental signals [1,2,3,4,5,6]. The defining feature of a TCS is the presence of a receptor (sensor) protein or protein complex, which controls the activity of a response regulator protein via phosphorylation (usually in presence of the signal, such as a particular molecule) or dephosphorylation (usually in the resting state). The best studied examples of TCS sensors are histidine kinases and chemoreceptors. Most of them are embedded in the membrane: only ~27% of histidine kinases [7], ~14% of bacterial chemoreceptors, and ~43% of archaeal chemoreceptors [8] were found to lack the transmembrane regions. The sensor domain of the transmembrane receptors is often located in the periplasm, and other domains are in the cytoplasm of the cell [5,9,10]. While many different architectures of TCS sensors are observed in nature, the most common ones share the organization of the membrane-proximal part, i.e., the transmembrane (TM) domain and the domains immediately adjacent to it [9,11,12,13,14]. Similar sensor domains are often utilized by histidine kinases and chemoreceptors, followed by a TM bundle consisting of four α-helices, and a cytoplasmic HAMP domain, a four-helical parallel coiled-coil [5,9,15]. Overall, HAMP domains are found in ~80% of chemoreceptors [8]; periplasmic sensor domains can be found in 21 out of 28 simple or hybrid transmembrane histidine kinases in *Escherichia coli* (str. K-12 substr. MG1655) listed in the MIST 3.0 database [16] using InterPro annotation [17]; 13 of these also have a cytoplasmic HAMP domain, whereas the only soluble histidine kinase in this strain does not have it. The role of the HAMP domain in these proteins is seemingly to transmit the signal and change its nature to the form that may be recognized by the downstream signaling domains such as DHp [9,12,15]. Consequently, similar ligand-induced conformational changes in the sensor, TM and HAMP domains might have been expected in different TCS sensors. Yet, several lines of evidence suggest different mechanisms in different signaling systems, and there is no definitive consensus in the field [9,12,13,18].

The difficulties in structural studies of TCS signal transduction arise from the size of the receptors, often reaching hundreds of angstroms in length, their dynamics and flexibility, and the transient nature of intermediate states and interactions with response regulator proteins. Moreover, whereas HKs are usually dimeric, chemoreceptors form trimers-of-dimers further organized in large arrays [19]. Up to this date, no atomistic structure of a full-length TM TCS sensor has been determined experimentally, although several models have been obtained in silico using homology modeling, low-resolution electron microscopy, and other data [20,21,22,23,24]. Consequently, the information about the details of signal transduction either comes from structural studies of isolated fragments of the receptors, indirect experimental data, or from molecular dynamics simulations. In particular, the crystallization of isolated periplasmic sensor domains and cysteine cross-linking studies were fruitful and led to several hypotheses of TM signaling [9] (in the absence of direct structural data). The (swinging) piston model posits that the signal is transduced via piston-like motions of the TM helices relative to each other [25,26,27,28]. The helical rotation model assumes that TM helices rotate during signaling [29,30]. Finally, diagonal scissoring models posit that the distances between alike TM helices (TM1-TM1 and TM2-TM2) change during signaling [12,31,32,33].

**Figure 1:**
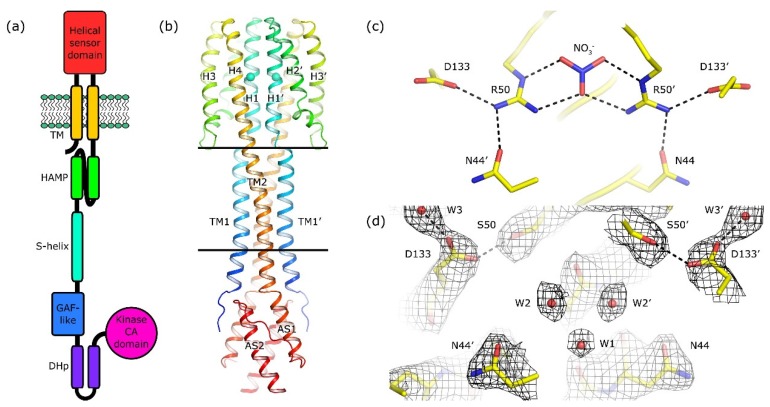
Architecture of NarQ and structure of the R50S mutant. Elements belonging to the second protomer in the dimer are marked with primes. (**a**) Architecture of NarQ (adapted from [9]). Note that the functional protein is homodimeric. Approximate domain boundaries, according to InterPro [17], are: TM1, residues 14–34; sensor, 39–146; TM2, 147–167; HAMP, 172–227; S-helix, 228–246; GAF-like, 247–360; DHp, 361–425; CA, 424–560. (**b**) Overall structure of the sensor-TM-HAMP fragment of the R50S mutant. Position of Ser50 is highlighted with spheres. The backbone structure is identical to that of the WT protein (Figure S1a). (**c**) Structure of the ligand-binding site in the WT protein. (**d**) Structure of the ligand-binding pocket in the R50S mutant. Asp133 is reoriented towards Ser50. 2F_o_−F_c_ electron density maps are contoured at the level of 1.2 × r.m.s. Putative water molecules are shown as red spheres.

Among the best studied TCSs are the nitrate/nitrite-sensing systems NarX-NarL and NarQ-NarP, responsible for the regulation of anaerobic respiration [34]. NarX phosphorylates NarL in the presence of nitrate and dephosphorylates NarL in its absence; NarQ phosphorylates both NarL and NarP in the presence of nitrate or nitrite and dephosphorylates both proteins in the absence of ligands [35]. Both NarQ and NarX consist of seven domains: a four-helical periplasmic sensor domain, TM bundle, HAMP domain, so-called signaling helix, GAF-like domain, dimerization and histidine phosphotransfer domain, and, finally, catalytic kinase domain (Figure 1a). Mutational studies highlighted the role of different amino acids in mediating the response of the proteins to nitrate and nitrite [36,37,38,39,40]. Whereas the sequence identity between NarQ and NarX is ~28%, their ligand binding sites—membrane-proximal parts of the sensor domain’s helices H1 called P boxes—are very well conserved: 14 out of 15 amino acids (residues 42–56 in NarQ) are identical [34], and the differing ones, Ile45 in NarQ and Lys49 in NarX, are responsible for the differentiation between nitrate and nitrite [36].

The structures of the sensor domains of NarX and NarQ have been determined. Cheung and Hendrickson determined the crystal structure of the NarX sensor domain both in the nitrate-free and nitrate-bound states [27]. We recently obtained the crystal structures of a larger fragment of NarQ, encompassing the sensor, TM and HAMP domains, also in the nitrate-free and nitrate-bound states, using the R50K mutant and the wild type protein, respectively [41]. These structures revealed the conformational changes in the TM bundle caused by binding of the nitrate ion to the sensor domain [41], namely diagonal scissoring, and twist and piston-like shifts, with the latter being responsible for relaying the signal to the HAMP domain [9].

Unexpectedly, the sensor-TM-HAMP fragment of NarQ displayed strong binding to its ligand nitrate, so we were not able to obtain the ligand-free structures of the WT variant [41]. Previous studies of NarX showed that replacing the arginine residue (Arg54, corresponding to Arg50 in NarQ) that coordinates the bound nitrate with lysine or serine resulted in loss of ligand sensitivity and locking of the receptor in the phosphatase state [36]. In accordance with these data, crystal structures of the R50K mutant of the sensor-TM-HAMP fragment of NarQ revealed the putative apo conformation of the receptor [41]. In the present work, we probe the effects of the R50S mutation as well.

Besides *E. coli* NarQ, several other proteins with similar nitrate sensor domains have been characterized in detail. Recently, Martín-Mora et al. determined the structure of the sensor domain PilJ of the *Pseudomonas aeruginosa* chemoreceptor McpN, which revealed a nitrate-binding site similar to that of NarQ and NarX [42]. Out of 15 NarQ H1 amino acids (42–56), seven are identical in McpN to those in NarQ, and four are similar. Replacement of Arg61, homologous to NarQ’s Arg50, with alanine rendered the protein unable to recognize nitrate [42]. Additionally, Boudes et al. determined the structure of the soluble transcription antiterminator protein NasR and found that its NIT domain is structurally similar to a dimer of NarQ/NarX-like four-helical sensor domains, with Arg50 and Arg176 forming a putative ligand-binding pocket [43]. In NasR, the replacement of either or both of Arg50 and Arg176 with alanine, serine, or lysine, locked the protein in the signaling state mimicking presence of nitrate [42]. However, the structural context of the nitrate-binding helical bundles in NasR is very different from that in transmembrane receptors, therefore we believe that the effects of mutations cannot be compared directly.

In this work we present the crystallographic structure of the R50S mutant of the sensor-TM-HAMP fragment and show that, surprisingly, the conformation of its backbone is identical to that of the backbone of the ligand-bound WT fragment, despite the absence of nitrate. We use molecular dynamics simulations to resolve this seeming contradiction and decipher the mechanism of NarQ activation at atomic level. We show that the ligand-induced conformational change in the ligand-binding site is helical rotation, which results in diagonal scissoring of the sensor domain helices, leading to the change in the secondary structure of the sensor-TM linker and, eventually, piston-like shifts of the TM α-helices. These results highlight our notion that these conformational changes are not mutually exclusive [9] and that care should be taken when analyzing the data resulting from the studies of truncated fragments [9,44].

## 2. Results

### 2.1. Crystal Structure of the R50S Mutant of NarQ

We obtained the crystals of the R50S mutant of the sensor-TM-HAMP fragment of NarQ using the in meso approach, similarly to our previous work on the wild type protein and its R50K mutant [41,44]. The crystals diffracted to ~2.0 Å, however the diffraction was anisotropic, and the final dataset was processed to the resolution of 3.3, 2.4, and 2.2 Å along the reciprocal lattice vectors. The crystals belonged to the same space group as the ligand-bound symmetric WT protein, F222. The data collection statistics are presented in Table 1.

We solved the phase problem using molecular replacement with the structure of the ligand-bound WT sensor-TM-HAMP fragment as a search model. Surprisingly, the backbone of the R50S mutant has the same structure as the ligand-bound WT fragment (Figure S1a), despite the Arg→Ser mutation and absence of nitrate in the ligand-binding site (Figure 1b–d and Figure S1b). In particular, there is a break in the α-helical structure between TM1 and the helix H1 of the sensor domain, whereas TM2 and the sensor domain helix H4 form a continuous α-helix (Figure 1b and Figure S1c). Overall, the root mean square deviation of the C_α_ atom positions between the WT (PDB ID 5IJI) and R50S structures is 0.2 Å.

### 2.2. Comparison of the Ligand-Induced Conformational Changes in NarQ and NarX

**Figure 2:**
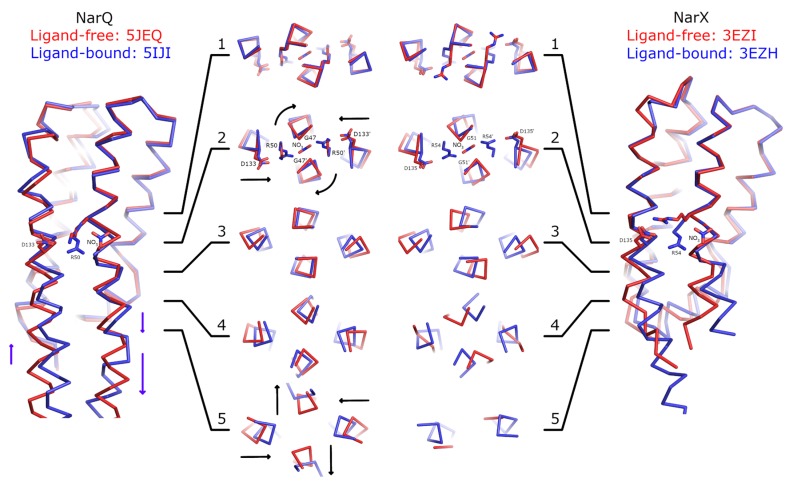
Comparison of ligand-induced conformational changes in NarQ and NarX. The structures are aligned using residues 48–54 and 122–130 (NarQ) or 52–65 and 124-132 (NarX). Nitrate, Arg50 and Asp133 (NarQ), and Arg54 and Asp135 (NarX) are shown explicitly. Binding of nitrate causes rotation of proximal glycine amino acids (Gly47 in NarQ and Gly51 in NarX) in the coiled coil layer 2. Slight displacement of the helices H4 is already apparent in the layer 2, and becomes very prominent in the layers 3–5. In NarQ, loss of α-helical structure in the H4-TM2 linker leads to more prominent piston-like motion of TM2 relative to TM1, as compared to the piston-like motion of H1 relative to H4, in coiled coil layer 5. Note that NarX constructs didn’t contain the TM domain and that the ligand-free NarX sensor domain (PDB ID 5EZI) is monomeric in crystal, with four chains in the asymmetric unit. The dimer was constructed manually by aligning chains A with chains A and B of the ligand-bound dimer. Second alternative conformation of Arg54 in ligand-free NarX is not shown as it is clearly incompatible with dimer formation.

To gain further insight into the mechanism of activation of nitrate receptors, we conducted a comparison of the ligand-induced conformational changes in the sensor-TM-HAMP fragment of NarQ [41] and in the sensor fragment of NarX [27] (Figure 2). We note that the ligand-bound sensor domain of NarX was crystallized as a dimer, whereas the ligand-free sensor domain of NarX was crystallized as a monomer [27]. Based on the observation that the sensor domains of NarQ in crystals (references [41,44] and this work) and in molecular dynamics simulations (this work) remain dimeric in the ligand-free form, we assumed that it is also the case for NarX, and constructed the dimer of the NarX sensor domain in the ligand-free form using the NarX sensor domain in the ligand-bound form as a template.

First and foremost, the comparisons reveal little structural alteration in the membrane-distant region of the sensor domain. Ligand-induced conformational changes are localized to the ligand-binding site and the membrane-proximal part of the sensor domain. As can be seen in Figure 2, the coiled coil layer 1, containing the backbone of Arg50 (NarQ) and Arg54 (NarX) is virtually unchanged by binding of the nitrate ion. Yet, in the layer 2, the following alterations are apparent: Gly47 (NarQ) and Gly51 (NarX) positions are affected, as the residues are rotated around the helix H1 axes, and helices H4 are slightly displaced towards the center of the coiled coil bundle. In the layers 3 and 4, diagonal scissoring becomes more apparent in NarQ, and very prominent in NarX. Finally, in the layer 5 of NarQ, ligand-induced break in the α-helical structure of the TM1-H1 linker becomes apparent, concomitant with motion of TM1 ends away from each other.

We note that in both NarQ and NarX sensor domains, the amplitudes of in-plane displacements of the α-helices H1 and H4 are larger and more prominent compared to the piston-like shifts. The piston-like shifts of TM1 relative to TM2 in NarQ are enhanced compared to the piston-like shifts of H1 relative to H4 by the break in TM1-H1 linker, from ~1 Å to ~3 Å for some of the residues. In NarX, the structure of the linker is not resolved, because the TM domain is missing in the crystallized construct.

### 2.3. Molecular Dynamics Simulations

**Figure 3:**
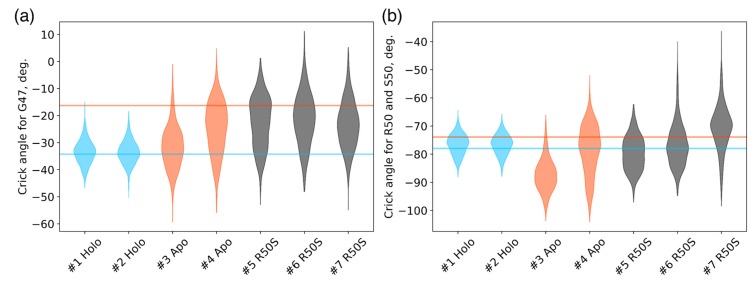
Crick angles for (**a**) Gly47 and (**b**) Arg50 or Ser50 in the last 200 ns of the simulations. Horizontal lines indicate the Crick angles in crystallographic structures, the values for the symmetric ligand-bound WT structure (PDB ID 5IJI) are shown in cyan, the values for the symmetric ligand-free WT structure (PDB ID 5JEQ) are shown in orange.

**Figure 4:**
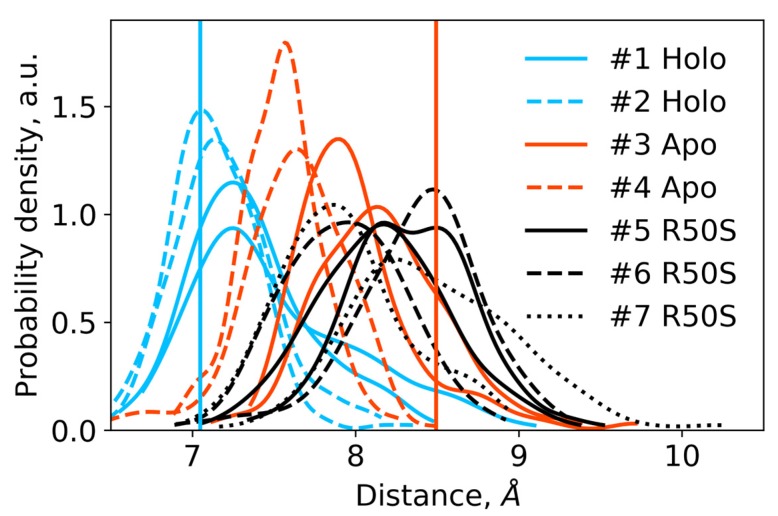
Distances between the backbone C_α_ atoms of the residues Arg50 or Ser50 and Asp133 within each protomer in the last 200 ns of the simulations. For each trajectory, two distributions are shown, with each one corresponding to a single protomer. The distances are indicative of intact hydrogen bonds between Arg50 and Asp133 in the WT protein and broken hydrogen bonds between Ser50 and Asp133 in the R50S mutant. Vertical lines show the respective distances in the crystallographic structures: the values for the symmetric ligand-bound WT structure (PDB ID 5IJI) are shown in cyan, the values for the symmetric ligand-free WT structure (PDB ID 5JEQ) are shown in orange.

**Figure 5:**
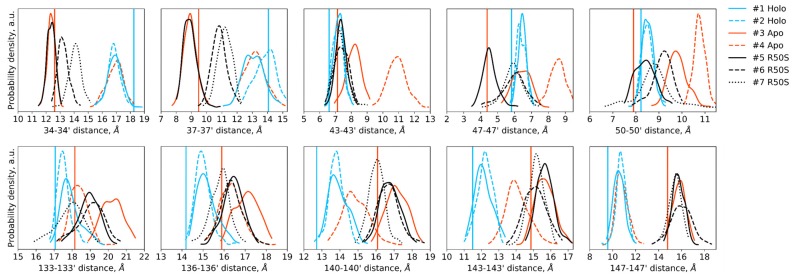
Distances between the backbone C_α_ atoms of the designated residues within the dimer in the last 200 ns of the simulations. Distances between the residues 34–50 of the two protomers are indicative of TM1-TM1′ and H1-H1′ distances, whereas distances between the residues 133–147 are indicative of H4-H4′ and TM2-TM2′ distances. Vertical lines show the respective distances in the crystallographic structures: the values for the symmetric ligand-bound WT structure (PDB ID 5IJI) are shown in cyan, the values for the symmetric ligand-free WT structure (PDB ID 5JEQ) are shown in orange.

**Figure 6:**
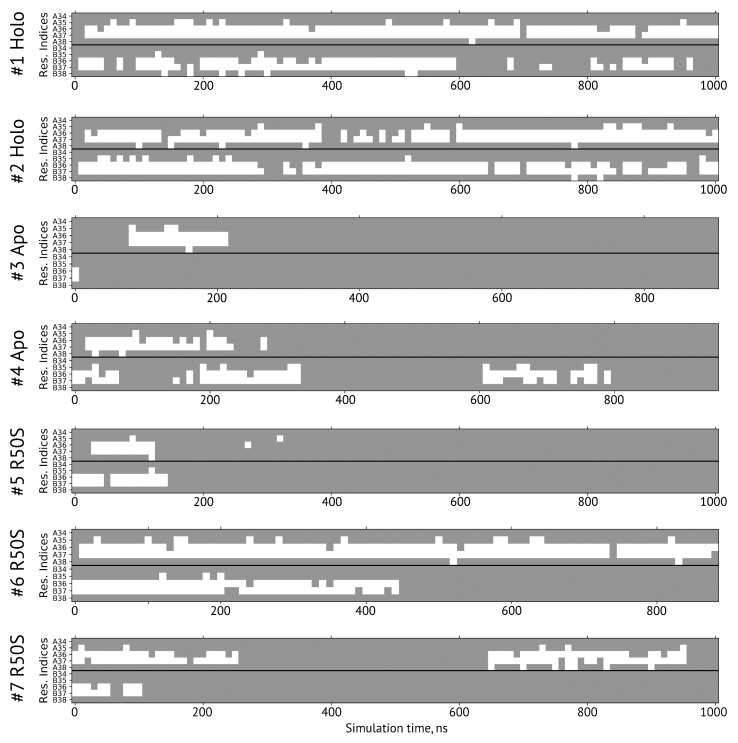
Secondary structure of the residues 34–38 forming the sensor-TM linker in simulations. A and B denote the two protomers in the NarQ homodimer. The residues with the φ and ψ angles characteristic of α-helical conformation are shown in gray, and those in disordered state are shown in white.

**Figure 7:**
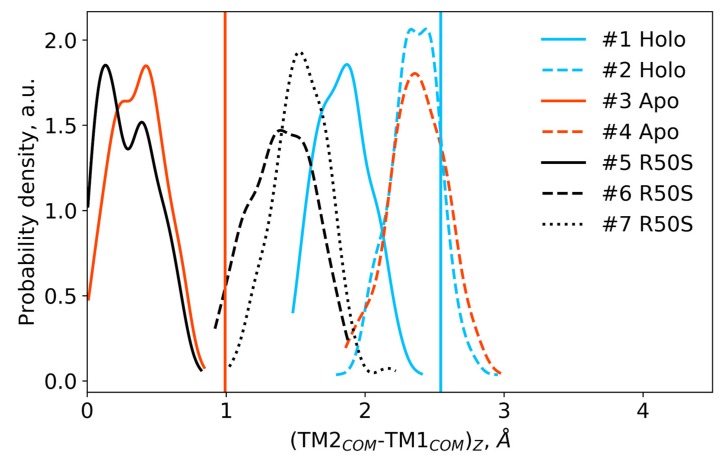
Difference in the projections of the TM1 and TM2 centers of mass on the z axis (normal to the membrane plane) in the last 200 ns of the simulations. Vertical lines show the respective piston shifts in the crystallographic structures: the values for the symmetric ligand-bound WT structure (PDB ID 5IJI) are shown in cyan, the values for the symmetric ligand-free WT structure (PDB ID 5JEQ) are shown in orange.

**Figure 8:**
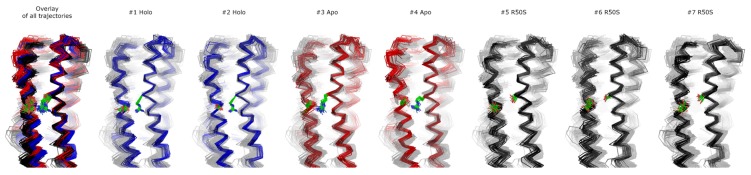
Structural snapshots from the last 200 ns of the simulations. 20 snapshots taken each 10 ns are shown. In each case, only one protomer is shown. Residues Arg50 or Ser50 and Asp133 are visualized as green sticks with oxygen and nitrogen atoms in red and blue, respectively. All other residues are visualized as ribbons. The structures were aligned using the residues 45 to 55 from both protomers. Individual MD trajectories may be found in the Supplementary Information.

To gain more insights into the nature of signaling-related conformational changes in the NarQ sensor and TM domains, we performed extensive molecular dynamics simulations of ligand-bound and ligand-free fragments as well as of the ligand-free R50S variant (Table 2). For the simulations #1-#6, the starting structure was based on that of the symmetric ligand-bound state that we determined earlier (PDB ID 5IJI, [41]), to avoid any potential bias that might result from differences in the starting structures, and also to see whether the switching of the sensor domain to the ligand-free conformation (previously obtained using crystallography [41]) may be observed in simulations. The ligand was removed in the ligand-free simulations, and Arg50 was replaced computationally with serine in the R50S simulations #5 and #6. We have also conducted the simulation #7 of the R50S mutant starting from the crystallographic structure of the mutant. Main observations are summarized in Figure 3, Figure 4, Figure 5, Figure 6 and Figure 7. Estimate of the asymmetry of simulated dimers [45] is presented in Figure S2. Structural snapshots from the last 200 ns of the simulations are shown in Figure 8; visualizations of the observed motions are presented in Supplementary Movies 1–7.

The ligand-bound systems (#1 and #2) conserved all of their essential features during the simulations. The ligand remained in the ligand-binding site throughout the trajectories. The Crick angles of Gly47 and Arg50 remained stable (Figure 3). The distance between Arg50 and Asp133 was largely conserved (Figure 4). No diagonal scissoring was observed, as reflected in the relative positions of H1 and H4 residues of the two protomers (Figure 5). The break between the helices TM1 and H1 persisted throughout the simulations (Figure 6), as did the relative shift between the pairs of TM1 and TM2 (Figure 7).

In the ligand-free systems (#3 and #4), we observed larger fluctuations of the Gly47 and Arg50 Crick angles, with the membrane-proximal ends of the helices H1 rotating relative to each other in the trajectory #3 (Figure 3). The distance between Arg50 and Asp133 C_α_ atoms (7.5–8.2 Å) was larger than in the ligand-bound systems (7.0–7.2 Å, Figure 4). H1-H1′ interhelical distances in the trajectory #3 were smaller compared to the trajectories #1 and #2, whereas H4-H4′ distances were larger (Figure 5), indicative of diagonal scissoring-like conformational change. In the trajectory #4, the interhelical distances were more similar to those observed in the ligand-bound structure (Figure 6). In both trajectories, TM1 and H1 formed a continuous α-helix for most of the simulations (Figure 7). A piston-like shift of the TM1 helices relative to TM2 was also observed in the trajectory #3 but not in the trajectory #4 (Figure 6).

Finally, in the ligand-free simulations of the R50S mutants (#5, #6, and #7), the Crick angles of Gly47 were similar to those of the ligand-free WT protein, whereas the Crick angles of Ser50 were more similar to those in the simulations of the ligand-bound WT protein (Figure 3). The hydrogen bond between Ser50 and Asp133 was broken, which resulted in larger distances between them compared to the WT protein, despite the serine’s side chain being shorter than that of arginine (Figure 4 and Figure 8). In all three of the simulations, H1 and H1′ were closer to each other, H4 and H4′ moved away (Figure 5), and TM1 and H1 formed a continuous α-helix in both protomers in the trajectory #5, in one of the protomers in the trajectory #6, and in one or both of the protomers in the trajectory #7 (Figure 6). Correspondingly, a piston-like shift compared to the ligand-bound WT variant was observed in all three of the simulations (Figure 7). Interestingly, simulations #6 and #7 also revealed partial dissociation of the membrane-distant parts of the sensor domains of the two protomers in parts of the trajectories (shown in Supplementary Movies).

## 3. Discussion

Several mechanisms of transmembrane signal transduction in TCS sensor proteins (HKs or chemoreceptors) have been proposed based on direct and indirect experimental and computational evidence. Yet, despite decades of studies, there is no consensus on whether these mechanisms are common for all TCS receptors.

Recently, we determined the crystal structures of the sensor-TM-HAMP fragments of a sensor HK NarQ that revealed the ligand-induced conformational changes in the TM and HAMP domains [41]. In particular, we observed that many different conformational changes happen concomitantly in the TM domain: diagonal scissoring at the periplasmic side, overall twisting of the TM bundle, and finally piston-like shifts of the TM helices [9]. In this work, we focused on signal generation in the sensor domain, and obtained new complementary data by crystallizing the R50S mutant of NarQ and simulating ligand-bound and ligand-free forms of the WT protein and the R50S mutant.

The results that we obtained highlight the strengths and weaknesses of the approaches that we employed. Whereas X-ray crystallography provides experimental data at high resolution, the obtained structures are static, and the details of conformational transitions cannot be grasped when the protein cannot be stabilized in intermediate conformations. On the contrary, molecular dynamics provides structural data with excellent time resolution, yet it is limited by the computational power available [46,47]. The timescales accessible to usual simulations are in the µs range, and many relevant biological processes are too slow to be captured in simulations at the moment [46,47].

Still, we obtained a reasonable correspondence between crystallography [41] and MD simulations (this work) for WT NarQ: the ligand-bound form retained all of its features throughout the simulations, whereas it converted into a ligand-free-like conformation in the simulations where the ligand was removed. In particular, in absence of ligands, Gly47 backbone oxygens lose the 3_10_-helix-like hydrogen bonds to Arg50 backbone nitrogen in the ligand-free simulations, which changes their Crick angles (Figure 3) and leads to helical rotation of H1 and restoration of a continuous α-helical structure between H1 and TM1 (Figure 6). Diagonal scissoring-like conformational change is also observed in the trajectory #3 (Figure 5), as are the piston-like shifts of the transmembrane helices (Figure 7). We believe that absence of diagonal scissoring and piston-like shifts in the trajectory #4 are the consequences of the limited length of the simulations, and would be observed if the simulations were extended using more computational resources. This belief is corroborated by the fact that in the R50S simulations (trajectories #5, #6, and #7, discussed below) diagonal scissoring and piston-like shifts are observed in all three trajectories. Finally, some deviations from idealistic behavior are expected since the systems are dynamic, and simulations might capture rare conformations prohibited by crystal packing or not detected in the averaged electron densities obtained in crystallographic experiments.

The data on the R50S mutant of NarQ helps us to elucidate the details of the mechanism of the transmembrane signaling by NarQ. Surprisingly, the mutant crystallized in the same conformation as the ligand-bound WT protein, despite the absence of nitrate ion in the mutant structure (Figure 1d). This observation appears to contradict the data that similar nitrate-binding-defective variants of related proteins NarX (R54S) and McpN (R61A) are locked in the inactive, ligand-free-like state [36,42]. MD simulations of the sensor-TM-HAMP fragment of the R50S mutant of NarQ reveal a fast transition to the ligand-free-like conformations, with all of its characteristic features such as diagonal scissoring and formation of a continuous α-helix between H1 and TM1. The Crick angles for Gly37 are similar to those of the ligand-free WT protein, yet, the Crick angles for Ser50 are closer to that of Arg50 in the ligand-bound WT protein. In the WT mutant, Arg50 forms a strong hydrogen bond with the nearby Asp133 under all conditions. A hydrogen bond between Ser50 and Asp133 is observed in the crystal structure of the R50S mutant (Figure 1d) but not in the MD simulations (Figure 4). Thus, Ser50 and Arg50 occupy the same conformation both in crystals and in simulations, whereas the rest of the protein differs, and position of the helix H4 is decoupled from the conformation of the residue 50 side chain. We conclude that the crystal structure of the R50S is partially artefactual, and the protein is trapped in the ligand-bound-like conformation by crystallization conditions or crystal contacts.

We also note that the overall equilibrium between the two conformations of the receptor must be finely tuned so that binding of the ligand can reliably shift the system from one state to another, and this equilibrium might be affected by artificial truncations used to crystallize the proteins, or indeed the inter-dimer contacts in crystals. Signaling proteins are often inherently strained and may populate several different states [12]. The sensor-TM-HAMP fragment that we used appears to have a much stronger nitrate binding constant as the ion could not be washed out from the protein [41]. Overall, using fragments for structural studies may be fruitful, but care should be taken while interpreting the resulting structures, especially the elements close to the truncation site [44].

Our results (Figure 1, Figure 3, Figure 4, Figure 5, Figure 6 and Figure 7) and reanalysis of the previously available data (Figure 2) allows us to postulate the mechanism of signal generation in nitrate sensors. In the ligand-free conformation, sensor helices H1 and H4 form continuous α-helices with TM1 and TM2, respectively. Binding of nitrate causes rotation of Gly47, which is in direct contact with it (Figure 2 and Figure 3), and leads to “helical” rotation of the membrane-proximal part of H1, accompanied by diagonal scissoring and eventually piston-like motions of the membrane-proximal ends of H1 and H4 ). Disruption of the α-helical structure of the H1-TM1 linker (Figure 2 and Figure 6) allows more flexibility in the relative position of TM1 and TM2, and leads to much more pronounced diagonal scissoring and piston-like shifts at the periplasmic side of the TM domain (Figure 2). In this mechanism, Gly47 plays a major role, along with Arg50, which coordinates the bound nitrate ion. In the ligand-bound structure, Gly47 is in direct contact with nitrate (Figure S3). Mutations of Gly47 to any other residue would result in a steric conflict between the C_β_ atom and the nitrate (Figure S3). Indeed, mutation of homologous Gly51 in NarX to arginine resulted in almost complete loss of nitrate sensitivity and reversal of the remaining effects of nitrate [36,48].

We note that the conformational changes in the sensor domain appear to be independent of the presence of the TM domain, because conformational changes observed in the separated sensor domain of NarX [27] are largely similar to those observed in the sensor-TM-HAMP fragment of NarQ (Figure 2). Conformational changes in other TCS proteins may be different: whereas NarQ has an α-helical sensor domain, many other receptors have mixed α/β sensor domains such as Cache; whereas NarQ binds its ligand at the dimerization interface, other proteins with α-helical sensors such as Tar [25] may bind the ligands peripherally and/or activate asymmetrically.

Our results provide a cautionary tale for attempts to infer the signaling-related conformational changes in the TM domain from the conformational changes in the isolated sensor domain. Changes in the α-helical structure of the TM1-H1 linker in NarQ (Figure 2 and Figure 6) could not be predicted from the structure of the isolated sensor domain yet they change the amplitude of the relayed conformational changes drastically. The data from the nitrate sensors show that helical rotation, diagonal scissoring and piston are simply different degrees of freedom in coiled coil proteins and are not mutually exclusive. Different parts of the signaling proteins may exhibit qualitatively different conformational changes, or no conformational changes at all, like the membrane-distant parts of the sensor domains (Figure 2). This is possible in part due to the fact that the α-helices forming the signal-transducing coiled coils at the core of TCS sensor proteins are not rigid: in NarQ, Gly47 alternates between α-helix and 3_10_-helix-like hydrogen bonding (Figure 2); H1-TM1 linker alternates between α-helical and disordered structure (Figure 2 and Figure 5); individual transmembrane helices bend around proline and glycine residues [9,49]. The linker between the TM and cytoplasmic domains may not necessarily be α-helical under all circumstances [50]. In the cytoplasm, signaling helix and DHp domains often bend asymmetrically in the available crystal structures [51,52,53,54,55,56]. In chemoreceptors, cytoplasmic coiled coil bundles are also flexible and have conserved glycine hinges [57,58]. Finally, in sensory rhodopsin transducers, cytoplasmic regions also reveal significant flexibility [59,60]. As the core-forming α-helices are relatively flexible by themselves, and often break, idealized mechanisms of signal transduction can be misleading, and care should be taken in interpreting indirect data. Overall, while structural studies of receptor fragments are often informative, direct data obtained using the intact full-length proteins is required for complete understanding of TCS signal transduction. With this in mind, and given that the receptors are often flexible, and their interactions with response regulator proteins are often transient, integrative structural biology approaches hold great potential for reconciling the data from different sources [20,21,22,23,24].

## 4. Materials and Methods

### 4.1. Cloning, Protein Expression, and Purification

The mutation R50S was introduced into the gene encoding the WT sensor-TM-HAMP construct using polymerase chain reaction. Expression and purification of the R50S variant were performed exactly as described previously [41]. In short, the protein was expressed in *E. coli* using an auto-inducing medium [61] and purified using metal affinity (Ni-NTA) and size-exclusion chromatography. Protein-containing fractions were pooled and concentrated to 30 mg/mL for crystallization.

### 4.2. Crystallization

The crystals were grown using the in meso approach [62,63,64], similarly to our previous work [41,44]. The solubilized protein in the crystallization buffer was added to the mono-oleoyl-formed lipidic phase (Nu-Chek Prep, Elysian, MN, USA). Crystallization trials were set up using the NT8 robotic system (LCP version, Formulatrix, Bedford, MA, USA). The crystals were grown at 22 °C and reached the final size of ~100 µm within 1 week. The crystals were obtained using the precipitant solution consisting of 1.2 M KH_2_PO_4_/Na_2_HPO_4_ pH 4.6. Before harvesting, the crystals were incubated for 5 min in the respective precipitant solutions supplemented with 20% glycerol. All crystals were harvested using micromounts (MicroLoops HT, MiTeGen, Ithaca, NY, USA), then flash-cooled and stored in liquid nitrogen.

### 4.3. Acquisition and Treatment of Diffraction Data

The diffraction data were collected at 100 K at the European Synchrotron Radiation Facility (ESRF) beamline ID23-1 [65] equipped with a PILATUS 6M-F detector (Dectris, Baden-Daettwil, Switzerland). The data collection statistics are reported in Table 1. In all cases, the diffraction was anisotropic as determined by decay of the CC_1/2_ values in 20° cones along the reciprocal cell directions using AIMLESS [66]. Diffraction images were processed using XDS (version from October 15th, 2015) [67]. STARANISO web server (version 3.315 released on February 12th, 2020) [68] was used to estimate the anisotropic resolution limits and to apply an anisotropic correction to the data. AIMLESS [66] was used as a part of the STARANISO protocol to merge and scale the data. Free-R labels were copied from the ligand-bound WT NarQ dataset (PDB ID 5IJI) [41].

### 4.4. Structure Determination and Refinement

The structure was solved using molecular replacement with MOLREP (version 11.2.08) [69] and the sensor and transmembrane domains from the structure of the sensor–TM–HAMP fragment (Protein Data Bank Identifier 5IJI) [41] as a search model. The model was refined manually using Coot (version 0.7.2) [70] and REFMAC5 (version 5.8.0073) [71]. The refinement statistics are summarized in Table 1.

### 4.5. Molecular Dynamics Simulations

In total, we conducted 7 MD simulations, listed in Table 2. In simulations #1–#6, the simulated systems (ligand-bound and ligand-free WT protein, ligand-free R50S mutant) were assembled using the crystal structure of the symmetric ligand-bound state (PDB ID 5IJI, [41]) as the starting point. In order to prepare models for the ligand-free and the ligand-free mutant, we removed the ligand and computationally replaced Arg50 with serine in the initial molecular model of the ligand-bound protein. In simulation #7, crystallographic structure of the R50S mutant was used as the starting structure. In all simulations, the protein was embedded in a model bilayer mimicking bacterial inner membrane composition consisting of 36 POPG and 108 POPE lipids with the lateral dimensions of 70.9 × 70.9 Å^2^ and then solvated with TIP3P water with a Na^+^/Cl^−^ concentration of 150 mM by means of the CHARMM-GUI web-service [72]. The simulation boxes had total dimensions of 70.9 × 70.9 × 164.0 Å^3^ and contained 78232, 78228, 78201 atoms for ligand-bound WT, ligand-free WT, and ligand-free R50S mutant system, respectively. All ionizable amino acids were modeled in their standard ionization state at pH 7.

The CHARMM-GUI recommended protocols were followed for the initial energy minimization and equilibration of the system. The atoms of protein and lipids in the system were subjected to a harmonic positional restraint and 5000 steps of steepest descent minimization followed by two 25 ps equilibration steps in the NVT ensemble using Berendsen thermostat and one 25 ps and three 50 ps equilibration steps in the NPT ensemble using Berendsen thermostat and barostat. During all of the equilibration steps, the force constants of the harmonic positional restraints were gradually reduced to zero. For the production simulations, Nose–Hoover thermostat and Parrinello–Rahman barostat were used. The temperature and pressure were set to 303.3 K and 1 bar with temperature and pressure coupling time constants of 1.0 ps^−1^ and 0.5 ps^−1^, respectively. All MD simulations were performed with GROMACS version 2018.6 [73]. The time step of 2 fs was used for equilibration simulations except for the early steps, while 5 fs was used for all production simulations allowed by the partial mass transfer from heavy atoms to the adjacent hydrogens. The CHARMM36 force field [74] was used for the protein, lipids, ligand, and ions. All production simulations were run in 2 replicates (see Table 2) ranging in the total simulation time from 880 to 1000 ns. The simulations were analyzed using custom scripts based on the MDAnalysis toolkit [75]. Crick angles were calculated as described by Strelkov and Burkhard [76].

## Figures and Tables

**Table 1:** Crystallographic data collection and refinement statistics.

**Data collection**	
Space group	F222
Cell dimensions	−
*a*, *b*, *c* (Å)	57.9, 73.7, 236.2
*α, β, γ* (°)	90, 90, 90
Wavelength (Å)	0.9724
Resolution (Å)	44.72–2.4 (44.72–6.8, 2.6–2.4) *
*R*_merge_ (%)	9.6 (3.0, 61.5) *
*R*_pim_ (%)	5.1 (1.8, 27.6) *
<*I*/σ*I*>	10.1 (21.3, 3.2) *
*CC1/2 (%)*	99.8 (99.9, 76.1) *
Completeness (spherical, %)	67.4 (99.0, 23.4) *
Completeness (ellipsoidal, %)	87.1 (99.0, 56.8) *
Multiplicity	4.6 (3.9, 5.7) *
Unique reflections	6860 (490, 490) *
**Refinement**	
Resolution (Å)	44.72–2.4
No. reflections	6′383
*R*_work_/*R*_free_ (%)	28.5/36.7
No. atoms	−
Protein	1829
Water	9
Average *B* factors (Å^2^)	−
Protein	39.2
Water	23.0
R.m.s. deviations	−
Protein bond lengths (Å)	0.003
Protein bond angles (°)	0.6
Ramachandran analysis	−
Favored (%)	98.3
Outliers (%)	0

* The data for the lowest and highest resolution shells are shown in parentheses. R.m.s.: root mean square.

**Table 2:** The list of performed simulations.

#	System	Trajectory Length, ns
1	Ligand-bound WT	1000
2	Ligand-bound WT	1000
3	Ligand-free WT	900
4	Ligand-free WT	950
5	Ligand-free R50S	1000
6	Ligand-free R50S	880
7	Ligand-free R50S	1000

## Supplementary Materials

Supplementary videos 1–7 show evolution of the structure of the sensor-TM-HAMP fragment of NarQ in molecular dynamics simulations with time. Each video corresponds to a different trajectory. Supplementary materials can be found at https://www.mdpi.com/1422-0067/21/9/3110/s1.

## Abbreviations

HAMP	Domain found in histidine kinases, adenylate cyclases, methyl accepting proteins and phosphatases
HK	Histidine kinase
MD	Molecular dynamics
r.m.s.	Root mean square
TCS	Two-component system
TM	Transmembrane
WT	Wild type
