# Supplementary material for: Sensor Histidine Kinase NarQ Activates via Helical Rotation, Diagonal Scissoring, and Eventually Piston-Like Shifts
Source: Int J Mol Sci. 2020 Apr 28;21(9):3110. doi: 10.3390/ijms21093110 (PMC7247690; doi:10.3390/ijms21093110)
Supplement: Supplementary file 1 [file ijms-21-03110-s001.zip › Supplementary Materials.pdf]

International Journal of
Molecular Sciences

MDPI

Sensor Histidine Kinase NarQ Activates via Helical Rotation, Diagonal Scissoring, and Eventually Piston-Like Shifts

Ivan Gushchin, Philipp Orekhov, Igor Melnikov, Vitaly Polovinkin, Anastasia Yuzhakova and Valentin Gordeliy

* Correspondence: ivan.gushchin@phystech.edu (I.G.), g.valentin@fz-juelich.de (V.G.)

Supplementary Information

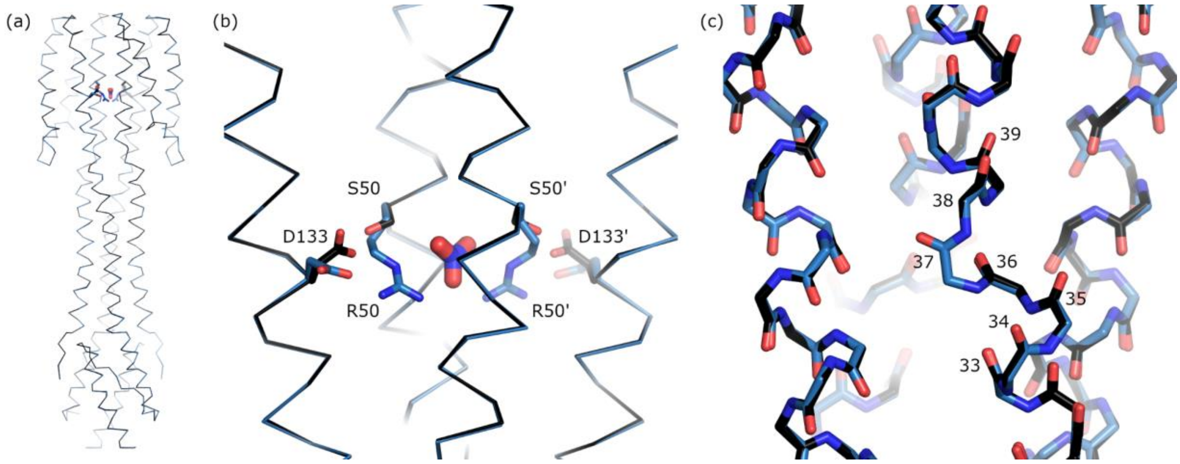

Figure S1. Detailed comparison of crystallographic structures of sensor-TM-HAMP fragments of ligand-bound WT NarQ (blue) and its R50S mutant (black). (**a**) Comparison of C_α atom positions of WT and R50S variants. The backbone positions are essentially identical. (**b**) Comparison of the ligand-binding pocket. Position of Asp133 side chain is different between the two structures. (**c**) structure of the TM1-H1 linker in the crystallographic structures of ligand-bound NarQ and its R50S mutant. Amino acid numbers are indicated in the figure. Residues up to 34 form a continuous α-helix with TM1; residues starting from 38 form a continuous α-helix with H1.

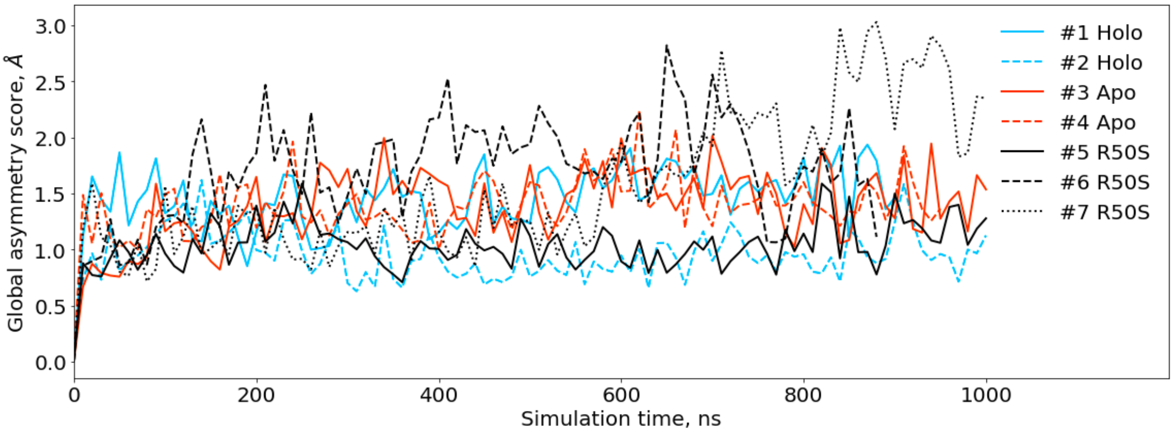

Figure S2. Estimate of the asymmetry of simulated sensor-TM-HAMP constructs using the metric introduced by Swapna et al. (Swapna, L.S.; Srikeerthana, K.; Srinivasan, N. Extent of Structural Asymmetry in Homodimeric Proteins: Prevalence and Relevance. *PLOS ONE* **2012**, *7*, e36688, doi:10.1371/journal.pone.0036688 [45]). Dimers with the score values < 1 are assumed symmetric, dimers with the score values < 3 have limited asymmetric organization, and the score > 3 corresponds to profound asymmetry (not observed in the present study).

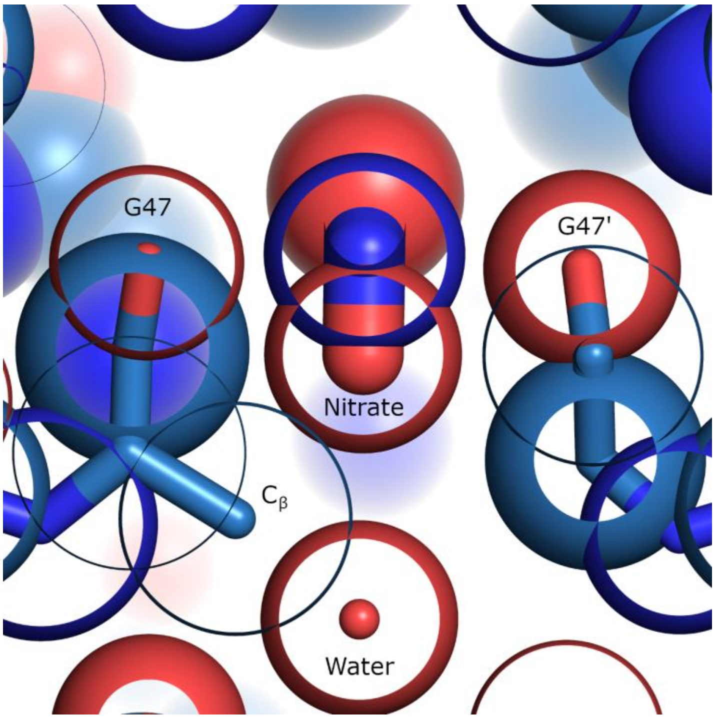

Figure S3. Interactions of the nitrate ion with glycine 47, and its interference with the putative position of the C_β atom in possible mutants of the glycine. Van der Waals radii of the nitrate and protein atoms are shown as spheres (cut for the atoms at the front). Mutation of Gly47 would result in displacement of the ordered water molecule beneath the nitrate ion, and would likely prevent the binding of the nitrate ion in this mode. Positions of the hydrogen atoms are not shown.
